# Prevalence of clinical mastitis and its associated risk factors among dairy cattle in mainland China during 1982–2022: a systematic review and meta-analysis

**DOI:** 10.3389/fvets.2023.1185995

**Published:** 2023-05-18

**Authors:** Shuiyun Chen, Huiying Zhang, Junjun Zhai, Honghai Wang, Xuelong Chen, Yanping Qi

**Affiliations:** ^1^Anhui Province Key Laboratory of Animal Nutritional Regulation and Health, Anhui Science and Technology University, Fengyang, China; ^2^Shanxi Province Engineering & Technology Research Center of Shanbei Cashmere Goats, Yulin University, Yulin, Shanxi, China; ^3^Daqing Agricultural and Rural Bureau, Daqing, Heilongjiang, China

**Keywords:** dairy cows, clinical mastitis, meta-analysis, prevalence, systematic review

## Abstract

**Background:**

Bovine mastitis is one of the most common and prevalent diseases affecting dairy cattle worldwide. It adversely affects the quality and quantity of milk production and leads to a significant economic loss for the farmers.

**Methods:**

This article aimed to estimate the prevalence of clinical mastitis (CM) infection in mainland China using a systematic review and meta-analysis. The research reports published during 1983–2022 in English or Chinese from databases (PubMed, Google Scholar, Cochrane Library, Science Direct, Web of Science, VIP Database for Chinese Technical Periodicals (VIP), Chinese National Knowledge Infrastructure (CNKI), and Wan Fang database) were identified after reviewing the relevant scientific literature. Based on our inclusion criteria, this study analyzed the prevalence of CM in 47 published studies prevalence extracted the total number of cattle infected with CM from the available studies, allowing us to estimate the prevalence of CM infection among these in mainland China.

**Results:**

The pooled prevalence with the 95% CI for the clinical mastitis was 10% (95% CI: 9.00, 12.00). The majority of CM was associated with lactation, parity, and age, with higher prevalence observed in late lactation 15% (95% CI: 11.00, 18.00) and mid-lactation 10% (95% CI: 6.00, 13.00) in comparison to early lactation 8% (95% CI: 5.00, 10.00). The incidence of CM increased significantly with the increase of parity and age, and the highest incidence rates were 19% (95% CI: 15.00, 23.00) and 16% (95% CI: 12.00, 19.00) at parity and age ≥7, respectively. Among the seasons, the highest prevalence of CM infection was found in autumn 9% (95% CI: 2.00, 17.00). Interestingly, no significant effects were evident regarding the influence of quarter on the prevalence of CM.

**Conclusion:**

Thus, estimating the prevalence of CM among cattle in mainland China. through meta-analysis can provide adequate measures to control CM, reduce economic losses, and prevent the spread and transmission of CM in Chinese herds.

## 1. Introduction

Mastitis is an inflammation of the mammary gland caused mainly by an infection with a wide range of bacteria (1); it is a severe disease that can adversely affect and commonly harms the dairy sector. It can also cause substantial economic losses to the dairy industry, such as lower milk value and reduced cow milk production, thus leading to the premature culling of cows (2, 3). Depending on the infection, mastitis can present in tinct forms: clinical mastitis (CM) and subclinical mastitis (SCM). Clinical mastitis (CM) originates as a sudden onset of varying degrees of redness, swelling, heat, and pain in the diseased milk quarter, resulting in a significant reduction in lactation, thinning and yellowing of milk, symptoms of the flocculent material, and elevated body temperature (4, 5). The primary pathogens causing CM include *Escherichia coli, Klebsiella* sp., *Staphylococcus aureus, Coagulase Negative Staphylococci* (CONS)*, Streptococcus agalactiae, Streptococcus uberis*, and other streptococci species (6). Season, Fecundity, lactation, nutritional conditions, environmental health, feeding management, and other factors have been strongly correlated with the disease (7).

According to the National Bureau of Statistics of China 2022 (https://data.stats.gov.cn), the country has 102.16 million head of cattle. Milk production is 39.32 million tons, while the prevalence of mastitis is ~60–70%, with clinical mastitis accounting for 21–23% of the total morbidity in dairy cattle, and dairy mastitis costs US$15–45 billion per year (8, 9). It has been reported that significant disparities exist between regions in China due to the lack of CM preventive and control strategies in some areas. For instance, from 1980 to 1989, the Lanzhou Institute of Chinese Veterinary Medicine and Academy of Agricultural Sciences recorded an average incidence of clinical-type mastitis of 33.41% in 32 dairy farms spread over 22 cities in China. Changchun (10), Shanxi (11), and Foshan (12) had detection rates for CM of 7.5, 11.69, and 35%, respectively. Interestingly, a study reporting 90,053 cases of clinical mastitis on 47 large dairy farms in 13 provinces in China showed that the incidence of clinical mastitis was associated with factors related to lactation period, quarter, and parity (13). These findings suggest that the incidence of CM varies considerably by region and characteristics and can cause significant economic losses and public health safety concerns; therefore, a comprehensive analysis of the prevalence and various risk variables associated with the onset of CM in Chinese cattle was conducted and this study may guide the prevention and control of the spread of the disease and may assist animal practitioners and researchers.

## 2. Methods

### 2.1. Basic country profile

China is located in eastern Asia, on the western coast of the Pacific Ocean. The total land area is about 9.6 million square kilometers, and the entire sea area is about 4.73 million square kilometers. The China altitude range is between 0 and 8,848, with national average precipitation of 695 mm in 2020 (http://www.gov.cn/xinwen/202102/09/content_5586383.htm).

### 2.2. Search strategy

We performed a systematic literature search using the following keywords: “Clinical Mastitis or Mastitis,” “prevalence” or “Epidemiology,” or “epidemiological study,” “Studies, Epidemiological,” or “Study, Epidemiological,” or “Studies, Epidemiologic,” or “Epidemiologic Study,” or “Study, Epidemiologic,” “incidence,” or “diagnosis,” or “Incidence Proportion,” or “Incidence Proportions,” or “Proportion, Incidence,” or “cows,” or “dairy cow,” or “dairy cows,” or “Cow, Bee,” or “Cow, Dairy,” “China” or “Chinese,” or “People's Republic of China,” or “Mainland China,” and variations and combinations of these terms and Systematic Review and Meta-analysis (PRISMA) guidelines for meta-analysis (14) was used to determine the overall prevalence of CM in dairy cows in China. The databases that were explored for finding the different published articles relevant to the prevalence of CM were the VIP Chinese Journal Database, China National Knowledge Infrastructure, Wanfang Data, and PubMed databases. Studies from different journal publications from 1983 to 2022 were searched for the prevalence of CM in China. We focused on articles published between January 1, 1983, and April 31, 2022, in English and Chinese by conducting a database search.

### 2.3. Data processing and filtration

Two investigators (HZ and SC) independently retrieved the data from the included studies using a standardized data collection form after thoroughly evaluating the prevalence of CM in the published studies. First author, year of publication, location, number of cows investigated, number of CM-positive cows, sample season, quarter or lactation or age of cows, and litter size were among the main information that was extracted from the various studies. In addition, the original articles were reviewed in detail, and references cited in the retrieved articles were searched again to trace additional relevant studies missed by the search. Any disagreements between the two researchers were resolved through discussion or arbitration with a third expert (YQ). The criteria for the selection of this study were as follows:

1. Chinese studies on the prevalence of clinical mastitis in dairy cows.
2. Sample size < 15 cows, too small to obtain reliable conclusions.
3. Sampling location within mainland China.
4. Use of animal study categories only, specifying sampling time, sample size, number of positive samples, prevalence, and study location.

### 2.4. Analysis of the study quality

The quality of the studies was evaluated according to the Grading of Recommendations Assessment, Development, and Evaluation (GRADE) (15).In brief, we determined the score for each of the following items, scoring one point when the following information was provided: Clearly describe the purpose of the study, clearly indicate the test method, divide the subjects into different subgroups, describe the sampling method in detail, and accurately analyze the relevant risk factors. When overall scores in analyzed articles reached 4–5, 2–3, and 0–1 points, they were categorized as high-quality, intermediate, or low-quality studies, respectively. The quality assessment of the included studies was undertaken independently by two reviewers (HW and XC) based on the content of the articles.

### 2.5. Statistical analysis

We calculated the pooled prevalence of CM in the cows of the selected studies based on a meta-analysis. Because of the significant heterogeneity in the included studies, the random effects model in Stata 12.0(Stata Corp. College Station, Texas) was used to generate the forest plots. The forest plot can depict the combined estimate and the 95% confidence interval (CI). The publication bias was assessed by using the Egger test and represented graphically by a funnel plot, which was employed to determine the outliers in the CM prevalence studies. The different factors investigated by us included: the year of publication (before 2012; 2012 or later) and the geographical region (North China, East China, Southwest China, Central China, Northeast China, Northwest China, and South China). Moreover, we also analyzed the various additional risk factors, which included cow age, parity, lactation period, quarter, and the sampling season.

## 3. Results

### 3.1. Search results and eligible studies

**Figure 1:**
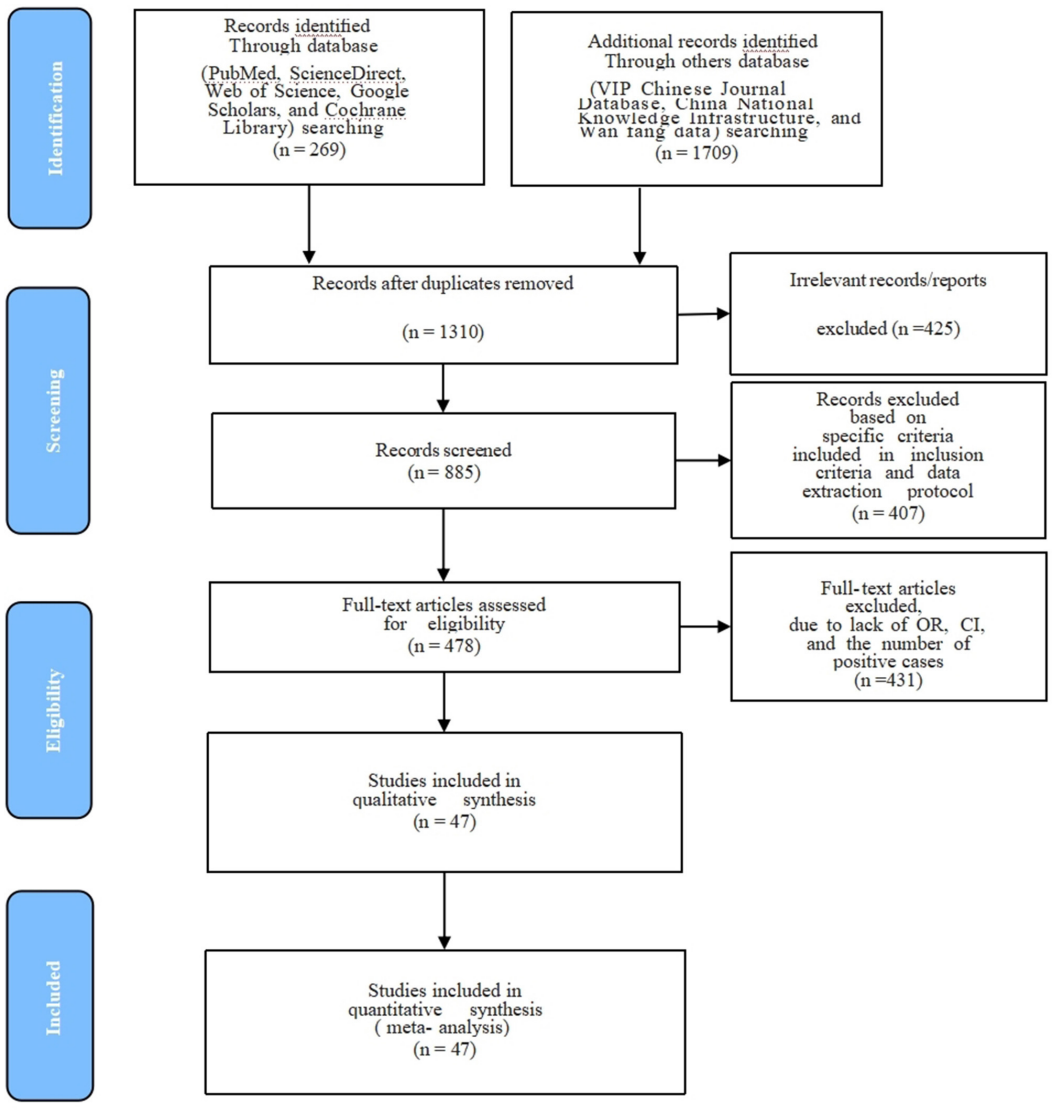
PRISMA flow diagram of the literature search, screening, assessing for eligibility, and selecting articles for the meta-analysis.

The present study aimed at employing systematic review and meta-analysis to estimate CM prevalence in the data extracted from studies related to China. The specifics used for the research included Chinese provinces, authors, years, and the reported prevalence of CM is presented (Table 1). All 47 were cross-sectional studies, 9 were of high quality (4 or 5 points), and 38 were of moderate quality (2 or 3 points). We have described in detail the procedures employed for screening articles and the reasons for exclusion, whereas articles displaying the precise prevalence of CM in China that were collected, were thoroughly examined, and considered for meta-analysis (Figure 1, Table 1). After excluding the duplicate citations and studies not relevant to this meta-analysis, a total of 1,657 full-text articles were included for screening, and a total of 47 articles met the inclusion criteria. We performed the present meta-analysis on the Chinese cattle herd and those included in the search were limited to period from 1983 to 2022 and covered 21 Chinese provinces. The meta-analysis included 13,924 CM positive cases out of 183,221 cattle (Table 2). It was evident that the Northwest region had the highest number of heads studied with 71,672; South China, was one of the seven with the lowest number of studies, nevertheless reported 135 positive cases.

**Table 1:** Included studies of CM infection in cattle in mainland China.

**References**	**Province**	**Region**	**Sampling time**	**No. examined**	**No. positive**	**Prevalence**	**Study design**	**Quality score**
Liu et al. (37)	Xinjiang	Northwest China	2019.05	240	35	15%	Cross sectional	2
Tao (38)	Sichuan	Southwest China	UN	90	5	5%	Cross sectional	2
Weng Chunling (39)	Heilongjiang	Northeast China	2,014	1,000	119	12%	Cross sectional	4
Song Jiaqi and Wenshuai (40)	Shandong	East China	UN	300	13	4%	Cross sectional	2
Wang Jinhe and Jun (41)	Henan	Central China	2009	24,257	1,742	7%	Cross sectional	3
Ma Fashun et al. (42)	Henan	Central China	2016.4–2017.3	500	73	15%	Cross sectional	4
Li Xiaonan et al. (43)	Inner Mongolia	North China	2,011	1,152	79	7%	Cross sectional	3
He (44)	Heilongjiang	Northeast China	UN	648	150	23%	Cross sectional	2
Liu Hong et al. (45)	Heilongjiang	Northeast China	2,009	390	17	4%	Cross sectional	2
Wang Dong et al. (46)	Ningxia	Northwest China	UN	2,527	333	13%	Cross sectional	2
Zhou et al. (47)	Heilongjiang	Northeast China	UN	3,576	639	18%	Cross sectional	5
Guo Junmei et al. (48)	Qinghai	Northwest China	2,016	2,029	87	4%	Cross sectional	4
Dai Min et al. (49)	Sichuan	Southwest China	UN	740	45	6%	Cross sectional	2
Zhou et al. (12)	Guangdong	Southern China	UN	387	135	35%	Cross sectional	2
Guohua (50)	Jiangxi	East China	UN	300	37	12%	Cross sectional	2
Wang Fushun et al. (51)	Tianjin	North China	2,012	500	71	14%	Cross sectional	2
Kang Lichao and Changbin (52)	Xinjiang	Northwest China	UN	300	34	11%	Cross sectional	2
Luo Jinyin et al. (53)	Gansu etc.	Northwest China	UN	2,173	115	5%	Cross sectional	2
Zhou Yanxuan et al. (54)	Chongqing	Southwest China	UN	118	10	8%	Cross sectional	2
Lulu (55)	Anhui	East China	2008.1–12	301	25	8%	Cross sectional	3
Yingying (56)	Henan	Central China	2016.5–12	420	47	11%	Cross sectional	2
Xiaohong (57)	Heilongjiang	Northeast China	UN	800	112	14%	Cross sectional	4
Wang et al. (58)	Guizhou	Southwest China	UN	175	58	33%	Cross sectional	2
Jinyin (59)	Gansu	Northwest China	2012.7–2013.8	344	19	6%	Cross sectional	2
Li (60)	Hebei	North China	UN	900	27	3%	Cross sectional	2
Jun (61)	Ningxia	Northwest China	2019.8–2020.7	59,953	1,120	2%	Cross sectional	2
Sanping (62)	Xinjiang	Northwest China	2013.2-2014.6	1,200	68	6%	Cross sectional	5
Yan (63)	Xinjiang	Northwest China	2017.12–2018.12	4,016	352	9%	Cross sectional	4
Haijun (64)	Jiangsu	East China	2010.4	1,000	116	12%	Cross sectional	5
Limei (65)	Jilin	Northeast China	UN	1,500	220	15%	Cross sectional	5
Wu et al. (66)	Beijing	North China	2001	144	7	5%	Cross sectional	3
Jin (67)	Shanxi	North China	UN	1,586	58	4%	Cross sectional	2
Zhou (68)	Hebei	North China	UN	1,150	130	11%	Cross sectional	3
Huizhen (69)	Shandong	East China	UN	760	53	7%	Cross sectional	2
Liu et al. (70)	Inner Mongolia	North China	UN	1,438	82	6%	Cross sectional	2
Li et al. (71)	Henan	Central China	1,983	78	3	4%	Cross sectional	5
Zhao et al. (10)	Jilin	Northeast China	2,005	1,000	75	8%	Cross sectional	2
Liu Xiwu (72)	Shandong	East China	2001.12–2002.12	315	29	9%	Cross sectional	2
Wang Bin et al. (73)	Shanghai	East China	UN	1,382	48	3%	Cross sectional	2
Ge (74)	Xinjiang	Northwest China	2,016	712	40	6%	Cross sectional	2
Zhongyi (75)	Shandong	East China	UN	257	23	9%	Cross sectional	2
Hao Jingfeng et al. (76)	Jilin	Northeast China	2,014	31,267	3,328	11%	Cross sectional	3
Chen Shenmiao et al. (77)	Shandong	East China	2008.1–2009.1	300	52	17%	Cross sectional	2
Han Wenru et al. (11)	Shanxi	North China	2,019	24,401	3,270	13%	Cross sectional	2
Ying (78)	Tianjin	North China	UN	1,9‘42	180	9%	Cross sectional	2
Jiahua (79)	Heilongjiang	Northeast China	UN	3,372	447	13%	Cross sectional	3
Liu Benjun et al. (80)	Heilongjiang	Northeast China	UN	1,281	196	15%	Cross sectional	2

UN, unclear.

**Table 2:** Pooled prevalence of CM infection in cattle in mainland China.

		**No. studies**	**No. tested**	**No. positive**	**% (95% CI)**	**Heterogeneity**		
						χ^2^	***P*** **-value**	*I*^2^ **(%)**
Region	Northeast China	11	45,182	53,22	12% (10–15)	30.79	0.000	96.7%
	North China	9	33,213	3,904	8% (5–11)	112.83	0.000	98.8%
	Northwest China	10	71,672	2,107	7% (5–10)	661.28	0.000	96.7%
	East China	12	6,022	446	8% (6–10)	305.11	0.000	90.3%
	South China	1	387	135	35% (30–40)	650.59		0.0%
	Central China	4	25,255	1,865	9% (5–13)	0.00	0.000	90.3%
	Southwest China	5	1,490	145	11% (6–17)	55.34	0.000	92.8%
Published time	>10 years/before 2012	23	40,999	3,220	10% (8–11)	597.70	0.000	96.3%
	< 10 years/2012 or later	24	1,42,222	10,704	10% (8–13)	6297.97	0.000	99.6%
Age	2	4	1,921	88	4% (1–7)	33.37	0.000	91.0%
	3	4	1,166	86	8% (2–13)	50.76	0.000	94.1%
	4	5	3,279	264	8% (4–12)	65.64	0.000	93.9%
	5	5	2,456	314	12% (6–19)	101.72	0.000	96.1%
	6	5	2,424	342	13% (9–17)	33.25	0.000	88%
	7	2	671	126	10% (8–25)	2.60	0.107	61.6%
	>7	6	2,237	377	16% (12–19)	29.18	0.000	82.9%
	Not found	37	1,69,022	12,327	10% (8–12)	6581.18	0.000	99.4%
Parity	1	16	14,945	616	5% (4–5)	53.23	0.000	71.8%
	2	15	13,323	852	7% (6–8)	83.30	0.000	83.2%
	3	15	13,507	858	8% (7–10)	83.77	0.000	83.3%
	4	15	11,691	1,202	11% (9–13)	126.95	0.000	89%
	5	15	9,323	1,127	13% (11–16)	114.93	0.000	87.8%
	6	9	5886	941	16% (13–18)	23.67	0.003	66.2%
	≥6	15	9,772	2,073	19% (15–23)	222.47	0.000	93.7%
	Not found	31	1,04,774	6,255	10% (8–12)	3829.78	0.000	99.2%
Lactation	Early lactation	7	1,558	119	8% (5–10)	21.85	0.001	72.5%
	Mid lactation	7	2,789	329	10% (6–13)	61.64	0.000	90.3%
	Late lactation	13	4,796	829	15% (11–18)	110.91	0.000	89.2%
	Not found	40	1,74,067	12,647	10% (8–12)	6636.43	0.000	99.4%
quarter	Left front	14	19,480	1,322	6% (4–7)	227.71	0.000	94.3%
	Left Rear	14	19,485	1,359	6% (4–7)	220.23	0.000	94.1%
	Right front	14	19517	1,333	6% (4–7)	219.66	0.000	94.1%
	Right rear	14	19,402	1,388	6% (5–8)	241.37	0.000	94.6%
	Not found	33	164829	11500	9% (7–11)	6128.26	0.000	99.5%
Season	Spring	8	33,532	1,762	8% (4–11)	986.54	0.000	99.3%
	Summer	3	16460	408	8% (3–14)	90.18	0.003	97.8%
	Autumn	4	29,478	2,233	9% (2–17)	1540.58	0.000	99.8%
	Winter	3	15,754	421	4% (2–7)	12.13	0.002	83.5%
	Not found	38	87,997	9,100	11% (9–12)	1830.29	0.000	98%
Total	47	1,83,221	13,924	10% (9–12)		0.000	99.4%

**Figure 2:**
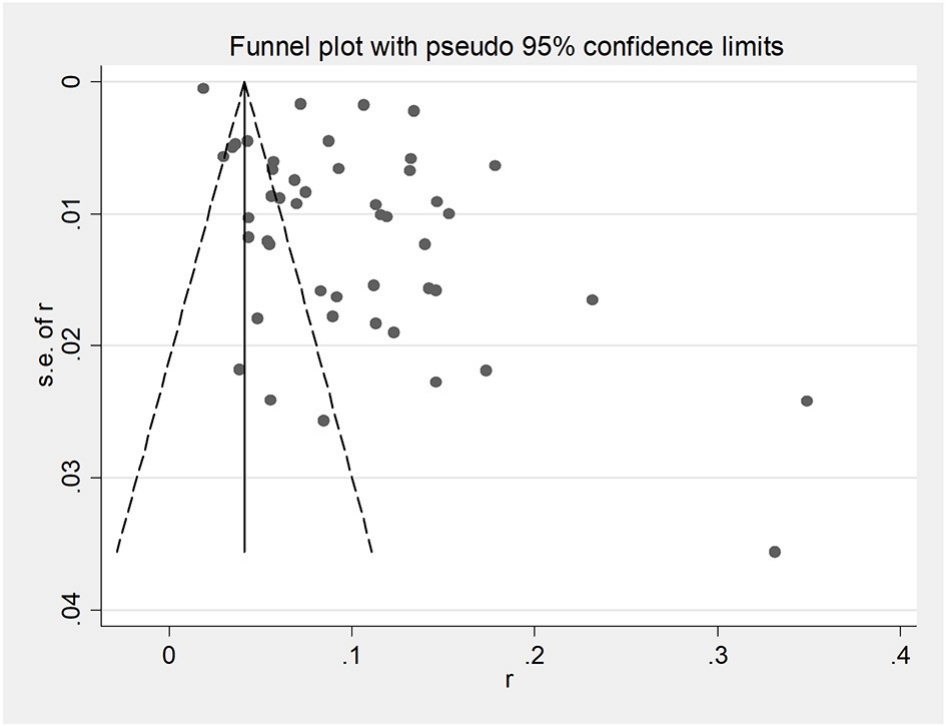
Funnel plot with pseudo 95% confidence limits for the analysis of potential publication bias.

**Figure 3:**
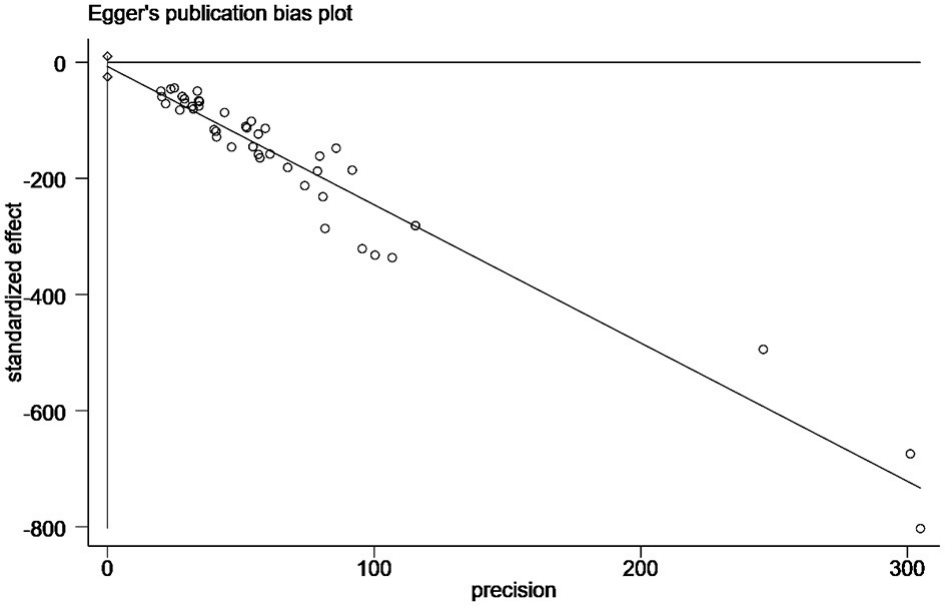
Egger's test for publication bias.

Funnel plots were used to assess potential publication bias in papers (Figure 2). Egger's test indicated a significant publication bias in this study (*P* < 0.05; Figure 3). However, as the finding was asymmetrical concerning overall prevalence, the results were potentially subject to publication bias.

### 3.2. Prevalence of CM in administrative regions or provinces in China

**Figure 4:**
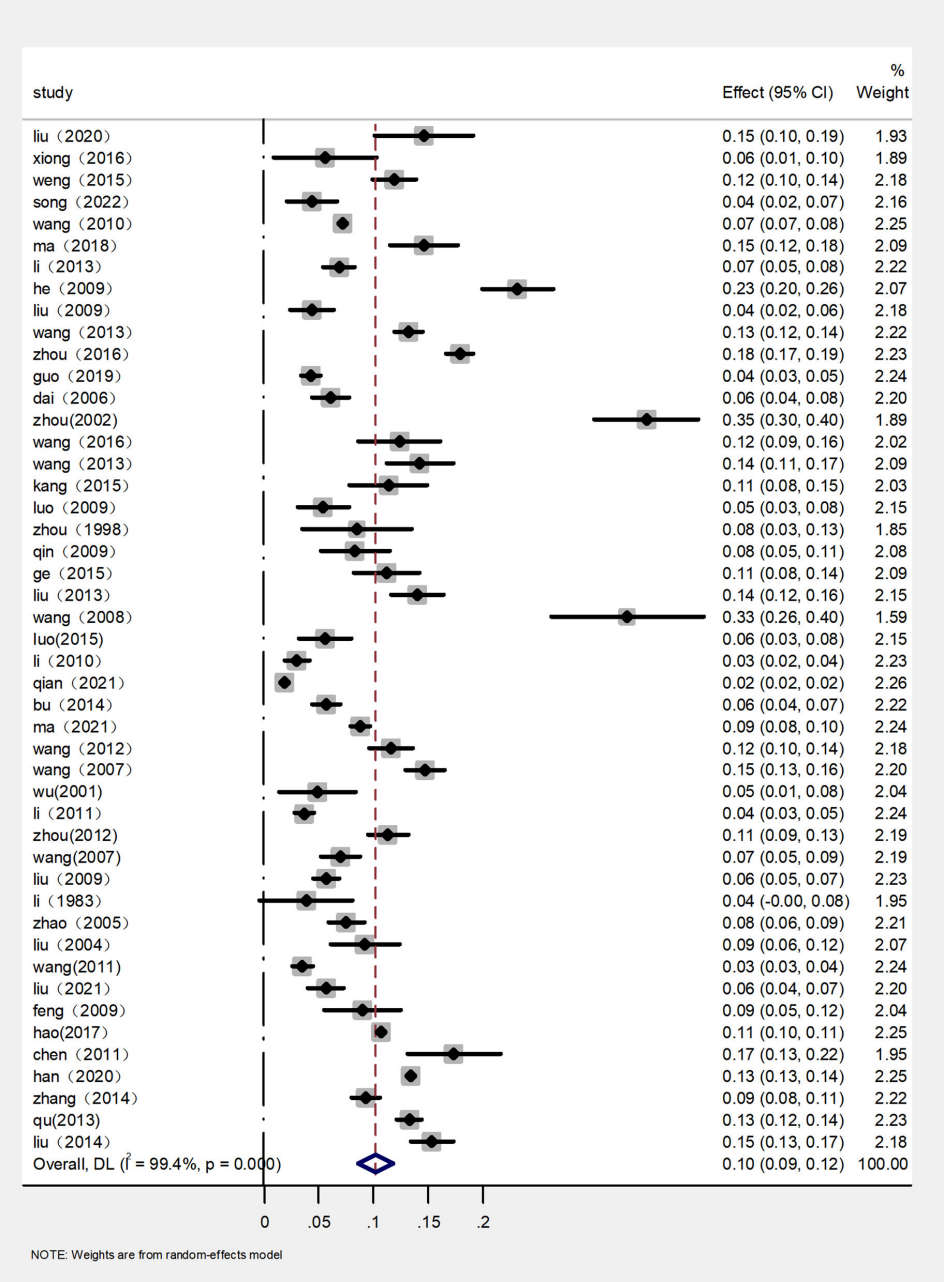
Random-effects meta-analysis of CM infections among cattle in mainland China.

**Figure 5:**
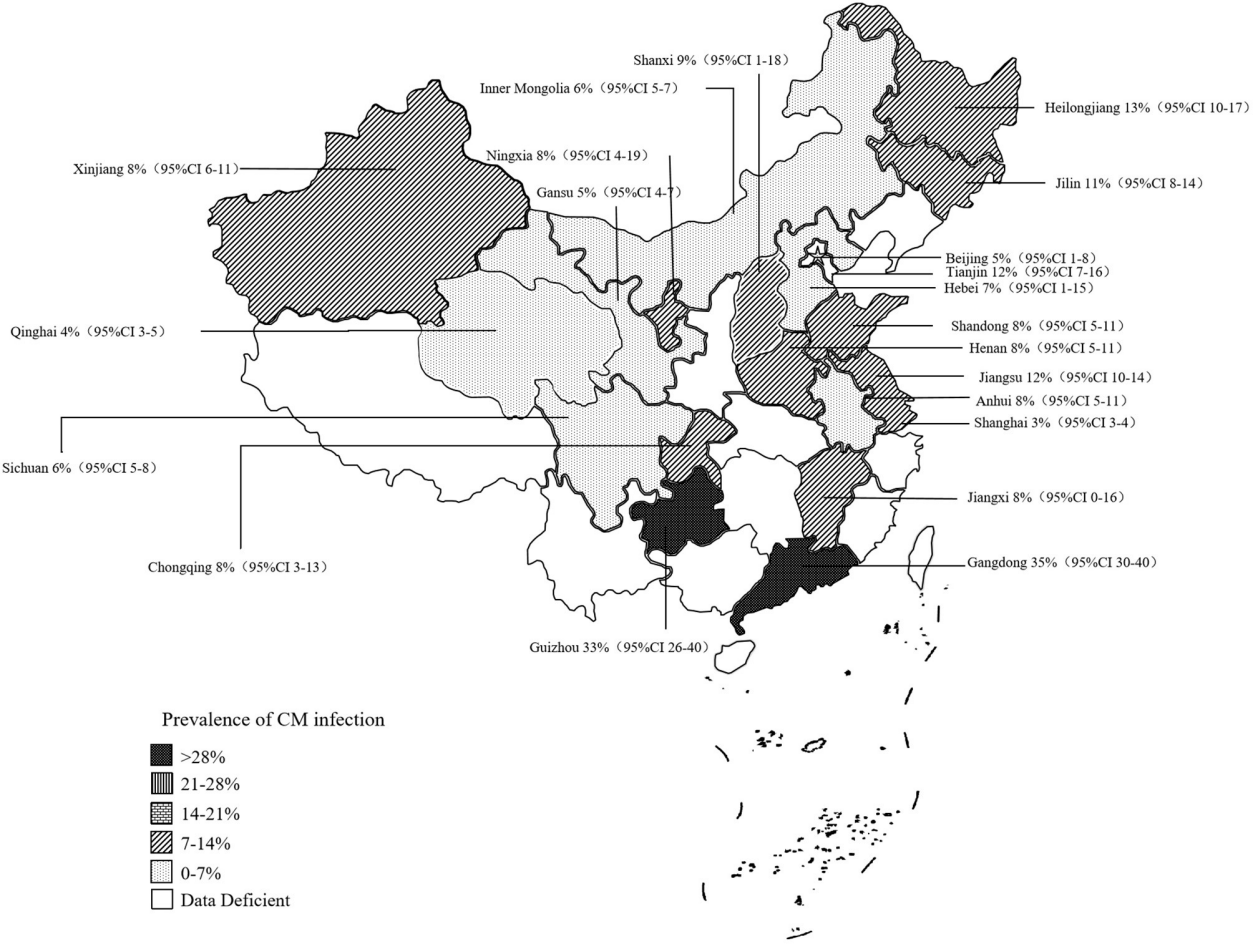
Map of CM infection.

The rates of prevalence of CM among cattle ranged from 2 to 35% (Figure 4, Table 1). The estimated prevalence of CM in the different provinces and regions in China was determined from 1,83,221 samples, and the combined prevalence of CM in cattle in China was estimated to be 10% (95% CI 9–12, 13,924/1,83,221) (Figure 5, Table 2). The prevalence of CM in Northeast China, Southwest China, Central China, East China, and North China were 12% (95% CI: 10.00,15.00, 5,322/45,182), 11% (95% CI: 6.00, 17.00, 145/1,490), 9% (95% CI: 5.00, 13.00, 1,865/25,255), 8% (95% CI: 6.00, 10.00, 446/6,022), 8% (95% CI: 5.00, 11.00, 3,904/33,213).In addition, it was found that the most studies on the epidemiology of CM in dairy cattle have focused on cattle farms in the northeast and northwest regions of China (Table 2). Despite fewer studies, the prevalence of CM in south China was as high at 35% (95 % CI 30–40, 135/387), which was significantly greater than that in northwest China, which had a prevalence of just 7% (95% CI 5–10, 2,107/71,672) (Table 2).

Based on the provinces-wise breakdown shown, the highest prevalence of CM was observed in Guangdong 35% (95% CI: 30.00, 40.00), followed by Tianjin 35% (95% CI: 30.00, 40.00), Guizhou 33% (95% CI: 26.00, 40.00), Heilongjiang 13% (95% CI: 10.00, 17.00), Jiangsu 12% (95% CI: 10.00, 14.00), Jilin 11% (95% CI: 8.00, 14.00); In Shanxi 9% (95% CI: −1.00, 18.00), Anhui 8% (95% CI: 5.00, 11.00), Henan 8% (95% CI: 5.00, 11.00), Jiangxi 8% (95% CI: 0.00, 16.00), Shandong 8% (95% CI: 5.00, 11.00), Xinjiang 8% (95% CI: 6.00, 11.00), Chongqing 8% (95% CI: 3.00, 13.00), Hebei 7% (95% CI: −1.00, 15.00), Inner Mongolia 6% (95% CI: 5.00, 7.00), Sichuan 6% (95% CI: 5.00, 8.00), Beijing 5% (95% CI: 1.00, 8.00), Gansu 5% (95% CI: 4.00, 7.00), and thus the overall prevalence of CM was < 10%. The prevalence of CM in other provinces was below 5%, which included the provinces of Qinghai 4% (95% CI: 3.00, 5.00) and Shanghai 3% (95% CI: 3.00, 4.00) (Figure 5, Table 3).

**Table 3:** Estimated pooled prevalence of Clinical mastitis by provincial regions in China.

**Province**	**No. studies**	**Region**	**No. tested**	**No. positive**	**Prevalence %**	**% (95% CI)**
Anhui (55)	1	Eastern	301	25	8	(5–11)
Beijing (66)	1	Northern	144	7	5	(1–8)
Chongqing (54)	1	Southwestern	118	10	8	(3–13)
Gansu (53, 59)	2	Northwestern	695	38	5	(4–7)
Guizhou (58)	1	Southwestern	175	58	33	(26–40)
Guangdong (12)	1	Southern	387	135	35	(30–40)
Hebei (60, 68)	2	Northern	2,050	157	7	(−1–15)
Heilongjiang (39, 44, 45, 47, 53, 57, 79, 80)	8	Northeastern	11,415	1,699	13	(10–17)
Henan (41, 42, 53, 56, 71)	5	Central	25,565	1,881	8	(5–11)
Inner Mongolia (43, 70)	2	Northern	2,590	161	6	(5–7)
Jilin (10, 65, 76)	3	Northeastern	33,767	3,623	11	(8–14)
Jiangxi (50, 53)	2	Eastern	737	56	8	(0–16)
Jiangsu (64)	1	Eastern	1,000	116	12	(10–14)
Ningxia (46, 61)	2	Northwestern	62,480	1,453	8	(−4 to 19)
Qinghai (48)	1	Northwestern	2,029	87	4	(3–5)
Shandong (40, 53, 69, 72, 75, 77)	6	Eastern	2,292	185	8	(5–11)
Shanxi (11, 67)	2	Northwestern	25,987	3,328	9	(−1 to 18)
Sichuan (38, 49, 53)	3	Southwestern	1,197	97	6	(5–8)
Shanghai (73)	1	Eastern	1,382	48	3	(3–4)
Tianjin (51, 78)	2	Northern	2,442	251	12	(7–16)
Xinjiang (37, 52, 62, 63, 74)	5	Northwestern	6,468	529	8	(6–11)

### 3.3. Risk factors associated with the prevalence of CM

The number of cows studied, as well as age, lactation, and parity stage of cows varied considerably between the selected studies which might have contributed to the wide variation in the prevalence of CM in different regions of China. The details of the influencing factors related to the prevalence of CM were obtained by meta-analysis, and data included the time of publication, age, season, parity, lactation, and quarter (Table 2). The prevalence of CM in studies published before 2012, 2012 or later was 10% but with different confidence intervals (95% CI: 8.00, 11.00) and (95% CI: 8.00, 13.00), respectively, as seen in the publication time. The total combined prevalence of CM was 10% both before and after 2012, but the number of studies after 2012 was about 3.6 times higher than before 2012. Thus, based on the age it could be concluded that the highest prevalence of CM was 16% (95% CI: 12.00, 19.00) in cows >7 years old followed by 13% (95% CI: 9.00, 17.00) in 6-year-old CM, 12% (95% CI: 6.00, 19.00), 10% (95% CI: 8.00, 25.00) for 7-year-old CM, 8% (95% CI: 2.00, 13.00) for 3-year-old CM, also 8% (95% CI: 4.00, 12.00) for 4-year-old CM and a minimum of 4% (95% CI: 1.00, 7.00) for 2-year-old CM. The studies related to the prevalence of CM by parity showed that the prevalence of CM was 5% (95% CI: 4.00, 5.00) for 1 gestation, 7% (95% CI: 6.00, 8.00) for 2 gestations, 8% (95% CI: 7.00, 10.00) for 3 gestations, and 11% or more for 4 gestations. The prevalence of CM in early lactation was found to be 8% (95% CI: 5.00, 10.00), mid-lactation was 10% (95% CI: 6.00, 13.00), and late lactation was (95% CI:11, 18). Based on the udder regions, it could be concluded that the prevalence of CM in all four different quarters was 6%, but the confidence intervals were (95% CI: 4.00, 7.00) for left rear, right front, right rear, and (95% CI: 5.00, 8.00) for the right posterior. The season was divided into four distinct periods: spring (March to May), summer (June to August), autumn (September to November), and winter (December to February). The prevalence of CM in spring, summer, autumn, and winter was 8% (95% CI: 4.00, 11.00), 8% (95% CI: 3.00, 14.00), 9% (95% CI: 2.00, 17.00), and 4% (95% CI: 2.00, 7.00), respectively (Table 2).

## 4. Discussion

Bovine mastitis remains a significant epidemic disease affecting cattle, with contrasting reports from the different production systems nationwide. CM infection is a severe threat to the health of dairy cows, thereby severely restricting the healthy development of the dairy industry and causing substantial economic losses to China's dairy industry. Therefore, this study presents a systematic evaluation and meta-analysis of the prevalence of CM in Chinese bovines that can be used as potential guideline for the strategic management of dairy farms, mastitis control, and reduction of economic losses.

To our knowledge, no systematic meta-analysis of national CM prevalence estimates and potential risk factors has been published previously. This study is the first meta-analysis of the pooled prevalence of CM in dairy cows in China. In addition, prior studies have indicated that CM is widely distributed almost across most of China, with an overall prevalence of clinical mastitis in dairy cattle of 10% in this study. This finding is consistent with CM prevalence in dairy cattle in Ethiopia 12.89%, Asella 10.3%, Ecuador12% (16–18) compared to Zimbabwe 3.8%, Brazil 2.3%, Kenya 6.8% (19–21), the prevalence of CM is significantly higher. In contrast, the prevalence of CM in this study was found to be lesser than 21.9% and 18% to that reported from Japan and India, respectively (22, 23), and this variation could be attributed to the differences in agro-climatic conditions and farm management practices (24).

We included studies from 7 different regions and 21 provinces in China; fewer studies were available for inclusion in a meta-analysis from South China which could be the reason for the high prevalence of CM compared to other regions. We recommend a more detailed survey of CM prevalence in these regions to provide a sound scientific basis for future prevention and control efforts. Furthermore, it was observed that the lowest preponderance was in Northwest China, where prevalence reached a minimum of around 7% among cows. According to the China Statistical Yearbook 2022 (http://www.stats.gov.cn/sj/ndsj/2022/indexch.htm), by the end of 2021, there was 21.286 million head of cattle in northwest China, thus accounting for 21.7% of the country. Thus, it could be speculated that the dairy cows in this region are kept in higher numbers in comparison to other areas and that the large-scale farms provided better preventive measures.

In this study, we found that the prevalence of CM in dairy cows was closely related to their age but showed a general increase with age. This result was consistent with the previous studies (25) and could be attributed to the fact that with age, immunity was relatively weaker, and disease-causing microorganisms are more likely to invade the cows. It is also possible that prolonged exposure to the milking apparatus leads to the loss of the normal teat terminal function and, thus, the development of CM. Therefore, farms can promptly cull the older cows to reduce the incidence of clinical mastitis.

In addition, we also observed that parity and lactation could also influence the prevalence of CM, with an increased risk of CM observed in cows with multiple parity in comparison to those with few parity and moderate parity. This finding was consistent with the previous reports published earlier (26, 27). This could potentially result from the prolonged calving and lactation of cows with multiple parity, thereby causing damage to the milking area, thus significantly increasing the chance of udder infection with pathogenic bacteria. Furthermore, the increased prevalence of CM in late lactation compared to early lactation was also consistent with the previously reported studies of mastitis (28–30), and relatively higher prevalence of mastitis in late lactation could be due to the repeated exposure to infection factors (cumulative infection) during milking lactation (31). Moreover, a number of previous studies have suggested that CM could be more prevalent in early lactation (26, 32). Consequently, teat massage and routine disinfection are recommended for multi-breed and lactating cows to significantly reduce the prevalence of CM by mitigating udder damage from prolonged milking.

Since 1983, there has been a steady stream of reports related to CM in China. We also found in this study that the highest infection rate of 9% in autumn, followed by 8% in spring and summer, respectively. Interestingly, one prior study has shown that CM infections tend to occur in July-September (33). Generally, the high CM season has been reported in the summer season because of the existence of high temperature, high humidity, and heat stress in the cows leading to loss of appetite and organism immunity, which can then accelerate excessive multiplication of disease microorganisms and result in high CM incidence (33). This difference was presumed to be due to the study's different sample areas and sampling seasons reported in different analyzed studies. Therefore, more attention should be paid to prevention during summer to impede the occurrence of CM. During the hot season, ventilation, shade, as well as dry and clean environment should be maintained in cattle sheds, occurrence of CM infection should be monitored, and infected cattle should be isolated promptly to prevent the CM onset. Owing to the meta-analysis being conducted on epidemiological surveys, microbiological investigations were not the objective of this investigation, it was not included in this analysis. However, based on the finding of the previous studies, *S. agalactiae* is a vital pathogen causing bovine mastitis in China (34). Notably, one prior study showed that *S. aureus* is a significant contributor to cow mastitis, whereas *E. coli* was the primary causative agent of CM (35). *S. aureus* is an infectious agent transmitted mainly during milking (36). We speculate that the high detection rate of large numbers of *S. aureus* could be due to improper milking practices resulting in the ease with which these organisms can be transmitted from infected to healthy sites via the contaminated milkers' hands, towels, or milking equipment. On the contrary, the high detection rate of *E. coli* could be attributed to the poor sanitary conditions in the cattle houses and playing fields, sewage sludge, fecal matter not being removed promptly, and this could be especially applicable to the cattle houses and playing fields where disinfection is not strict or not carried out, thereby resulting in bacteria entering the udder through the teat.

This meta-analysis provides an overview of CM profiles in China. The findings clearly revealed significant differences in CM prevalence across provinces and regions, but we must admit that this study has few limitations. First, 47 pieces of data reported in this study was obtained from 8 large databases, but some relevant studies may still need to be included. Secondly, the sample size of some studies was small, and limited information was included, which may lead to unstable overall estimation and subgroup analysis results. Third, only one survey or investigation was conducted in some provinces, which might not accurately represent the true prevalence of CM in these areas. Finally, due to the lack of available literature, various potential risk factors such as breed, history of previous mastitis, floor type, and breeding environment of dairy cows were not analyzed. However, this meta-analysis can provide novel insights about overall prevalence and development trend of CM infection in China during the survey period.

In conclusion, this study showed that the overall prevalence of clinical mastitis in Chinese dairy cows was 10%, with the highest and lowest prevalence of CM in South China and Northwest China being 35 and 7%, respectively, regarding the region. Parity, age, season, and lactation of Chinese cows are potential risk factors for mastitis. It is recommended that relevant practitioners improve management strategies for disease to develop appropriate prevention and control programs. In addition, monitoring of CM should be strengthened, and prevention and control programs should be adjusted promptly according to infection, which may help reduce CM's prevalence in China.

## Data availability statement

The original contributions presented in the study are included in the article/supplementary material, further inquiries can be directed to the corresponding authors.

## Author contributions

Conceptualization: HW. Data collection: HZ, SC, and YQ. Writing—original draft preparation: SC. Data analysis: XC. Writing—review and editing: XC and YQ. Funding acquisition: JZ. All authors have read and agreed to the published version of the manuscript.
